# Examining guidelines and new evidence in oncology nutrition: a position paper on gaps and opportunities in multimodal approaches to improve patient care

**DOI:** 10.1007/s00520-021-06661-4

**Published:** 2021-11-23

**Authors:** Carla M. Prado, Alessandro Laviano, Chelsia Gillis, Anthony D. Sung, Maureen Gardner, Suayib Yalcin, Suzanne Dixon, Shila M. Newman, Michael D. Bastasch, Abby C. Sauer, Refaat Hegazi, Martin R. Chasen

**Affiliations:** 1 Human Nutrition Research Unit, Department of Agricultural, Food and Nutritional Science, University of Alberta, Edmonton, AB Canada; 2 Department of Translation and Precision Medicine, Sapienza University of Rome, Rome, Italy; 3 Peri Operative Program, McGill University Health Center, Montreal, QC Canada; 4 Department of Medicine, Division of Hematologic Malignancies and Cellular Therapy, Duke University School of Medicine, Durham, NC USA; 5 Florida Cancer Specialists and Research Institute, Fort Myers, FL USA; 6 Department of Medical Oncology, Hacettepe University Cancer Institute, Sihhiye, Ankara, Turkey; 7 Humana Healthcare Research, Portland, OR USA; 8 Thompson Cancer Survival Center, Knoxville, TN USA; 9 University of Texas Health/East Texas Cancer Institute, Athens, TX USA; 10 Scientific & Medical Affairs, Abbott Nutrition, Columbus, OH USA; 11 Department of Medicine, University of Toronto, Toronto, Canada; 12 William Osler Health System, Brampton, ON Canada; 13 Department of Family Medicine, McMaster University, Hamilton, ON Canada

**Keywords:** Malnutrition, Low muscle mass, Nutrition, Exercise, Multimodal, Multidisciplinary

## Abstract

Malnutrition, muscle loss, and cachexia are prevalent in cancer and remain key challenges in oncology today. These conditions are frequently underrecognized and undertreated and have devastating consequences for patients. Early nutrition screening/assessment and intervention are associated with improved patient outcomes. As a multifaceted disease, cancer requires multimodal care that integrates supportive interventions, specifically nutrition and exercise, to improve nutrient intake, muscle mass, physical functioning, quality of life, and treatment outcomes. An integrated team of healthcare providers that incorporates societies’ recommendations into clinical practice can help achieve the best possible outcomes. A multidisciplinary panel of experts in oncology, nutrition, exercise, and medicine participated in a 2-day virtual roundtable in October 2020 to discuss gaps and opportunities in oncology nutrition, alone and in combination with exercise, relative to current evidence and international societies’ recommendations. The panel recommended five principles to optimize clinical oncology practice: (1) position oncology nutrition at the center of multidisciplinary care; (2) partner with colleagues and administrators to integrate a nutrition care process into the multidisciplinary cancer care approach; (3) screen all patients for malnutrition risk at diagnosis and regularly throughout treatment; (4) combine exercise and nutrition interventions before (e.g., prehabilitation), during, and after treatment as oncology standard of care to optimize nutrition status and muscle mass; and (5) incorporate a patient-centered approach into multidisciplinary care.

**Supplementary Information:**

The online version contains supplementary material available at 10.1007/s00520-021-06661-4.

## Introduction

Malnutrition, muscle loss, and cachexia are prevalent in patients with cancer and associated with poor outcomes, regardless of body weight or body mass index (BMI). Approximately 70% of patients with cancer develop malnutrition [1]. Low muscle mass is a phenotypic criterion of malnutrition affecting approximately 40% of patients and may be caused by reduced intake, low levels of physical activity, effects of cancer, and/or anticancer therapies [2, 3]. Low muscle mass also occurs in cachexia (with or without loss of fat mass), a multifactorial wasting syndrome occurring in 50 to 80% of patients [4]. These conditions can occur before diagnosis, as well as during or after treatment [3, 5]. If untreated, they are associated with reduced physical function and quality of life, dose-limiting toxicities, reduced treatment response, increased risks for surgical complications, and reduced survival [3, 6–10]. In addition, they strain healthcare and economic resources, extending hospital length of stay (LOS) and increasing risks for unplanned hospitalizations and readmissions [10, 11].

Ensuring access to nutrition resources is fundamental to quality care. Early and proactive nutrition care – consisting of screening, assessment, and intervention – is associated with improved outcomes as patients progress through their cancer journey to cure or palliation [12–15]. A multidisciplinary team (MDT) approach to nutrition care is associated with mitigating the sequelae of malnutrition, muscle loss, and cachexia and improving outcomes [16].

A panel of international multidisciplinary experts in oncology and nutrition, exercise, and internal and family medicine participated in a virtual scientific roundtable in October 2020 to discuss gaps and opportunities in oncology nutrition care relative to international societies’ recommendations and current scientific evidence. The goals of this position paper are to (1) raise awareness around the lack of access to comprehensive nutrition care as an obstacle to optimizing patient outcomes, (2) increase understanding that nutrition interventions alone and in the context of multimodal care and delivered using MDT approaches increase the efficacy of anticancer therapies to improve outcomes, (3) highlight new evidence relative to current guideline categories of screening, assessment, intervention, and monitoring, and (4) provide clinical practice principles to optimize nutrition care in oncology.

## The combined burden of malnutrition and low muscle mass in cancer

Malnutrition (undernutrition) can result from inadequate intake and/or uptake of nutrients that can cause muscle mass loss, leading to reduced physical function and impaired clinical outcomes [17]. The prevalence of malnutrition is higher and more severe among older patients and those with upper gastrointestinal (GI), head and neck, and lung cancers than among patients with other cancer types [18]. The burden of malnutrition can be compounded by co-occurring low muscle mass in patients who are under- or overweight. More than 50% of newly diagnosed cancers are in patients with BMIs ≥ 25.0 kg/m^2^, among whom more than 60% can be at nutritional risk or malnourished [2, 19].

Low muscle mass (also referred to as sarcopenia) is a central feature of cancer. Sarcopenia in cancer is often secondary (disease-related), not primary (age-related), though the presence of age-related cachexia can be exacerbated by a cancer diagnosis [20]. Secondary sarcopenia has predominantly focused on muscle mass loss without consideration of muscle function, and the terms low muscle mass or myopenia may be used to avoid confusion with primary sarcopenia’s diagnostic criteria [21, 22]. Although malnourished patients can have low muscle mass, malnutrition may be present without myopenia or sarcopenia.

In addition to locating tumors to assess treatment response, computed tomography (CT) can be used to detect low muscle mass and the degree of fatty infiltration in muscle (myosteatosis). The relationship between low muscle mass and outcomes in patients with cancer is well documented [10, 23, 24]. A pooled analysis of skeletal muscle mass in patients with colorectal cancer (*n* = 215) found that low muscle mass was an independent predictor of poor treatment response and progression-free survival [23]. Similarly, a meta-analysis of 70 studies of patients with GI tumors (*n* = 21 875) demonstrated that preoperative low muscle mass was associated with increased risks for surgical complications and mortality [24]. Myosteatosis is an indicator of muscle quality and a predictor of poor patient outcomes [10, 25]. An analysis of 1630 preoperative patients with stages I to III colon cancer found increased hospital LOS, mortality rate, and postoperative complication risk among individuals with low muscle mass and/or myosteatosis compared with patients with adequate muscle mass and quality [10]. Notably, ultrasound is an additional tool that can measure muscle mass and quality (by echogenicity); however, its use in oncology has not been fully explored.

Many patients develop cachexia (also referred to as cancer-associated malnutrition), a wasting syndrome that cannot be fully reversed by conventional nutrition interventions (i.e., additional protein and energy). It is characterized by anorexia and systemic inflammation, which create negative protein and energy balance, leading to weight loss (with or without fat loss) and muscle wasting [26]. Cachexia can worsen with anticancer treatment and has severe negative consequences on quality and length of life [26]. Additionally, it can be present in patients with excess weight and is frequently underrecognized in this population [27]. Among 1473 patients with GI or respiratory tract cancers, Cox proportional hazard modeling demonstrated the combination of weight loss and low muscle mass and quality was associated with reduced survival across BMI categories. [27]. Interestingly, weight stability may mask changes in muscle quantity and quality. In a large sample of patients with early stage colorectal cancer (*n* = 1026), Brown et al. showed that despite weight stability, one in eight patients developed low muscle mass and one in seven developed myosteatosis during a 15-month follow-up period [28].

## Nutrition care practice: recommendations and current evidence

**Fig. 1:**
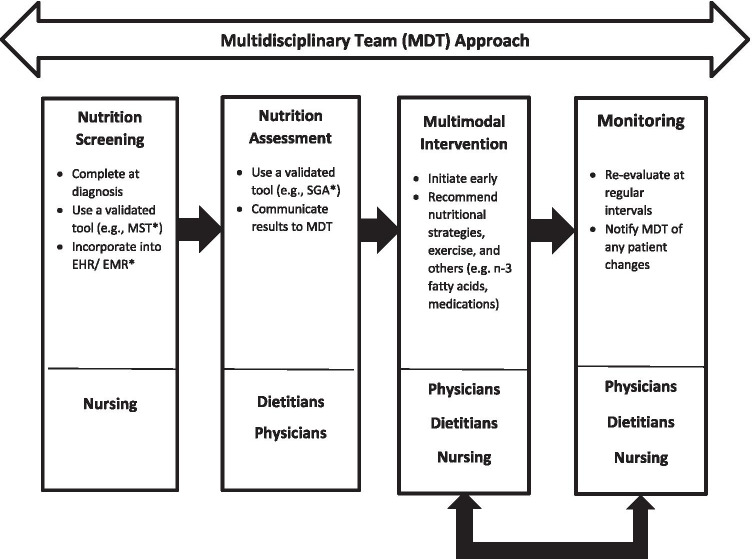
Proposed nutrition care process for oncology. *MST = Malnutrition Screening Tool; EHR/EMR = Electronic Health Record/Electronic Medical Record; SGA = Subjective Global Assessment

The goals of nutrition therapy are to maintain or improve energy and protein intake, mitigate metabolic abnormalities, preserve physical function, reduce the risk of treatment intolerance, and improve quality of life before, during, and after curative or palliative treatment [29]. The nutrition care process is a systematic way of providing nutrition care to patients across healthcare settings and is used to address these goals, which is an important aim of the MDT care approach (Fig. 1) [30].

Not all patients have access to comprehensive nutrition care, in particular when dietitian staffing is insufficient, reimbursement for nutrition services is absent, or screening standards are lacking. In a survey of cancer survivors (*n* = 1073 responses), fewer than 40% of patients with involuntary weight loss reported being seen by a dietitian during treatment [31]. Trujillo et al. reported that the average dietitian-to-patient ratio in outpatient cancer centers in the USA was 1:2308, far below the estimated ratio of 1:120 needed to provide proactive nutrition care and highlighting the need to expand nutrition resources and improve reimbursement [32].

Many international nutrition societies recognize the importance of nutrition as essential to comprehensive, high-quality oncology care. Societies, including the American Society of Clinical Oncology (ASCO), the American Society of Parenteral and Enteral Nutrition (ASPEN), the European Society for Clinical Nutrition and Metabolism (ESPEN), have published recommendations regarding nutrition care to guide clinical practice and improve patient and healthcare outcomes (Table 1).

**Table.1:** Nutrition care process – expert organization recommendations

Expert society	Malnutrition screening	Nutrition assessment	Nutrition intervention	Exercise	Multimodal intervention	Monitoring	Multidisciplinary team
Nutrition							
Academy of Nutrition and Dietetics (AND)	X	X	X			X	X
American Society for Parenteral and Enteral Nutrition (ASPEN)	X	X	X				
European Society for Clinical Nutrition and Metabolism (ESPEN)	X	X	X	X	X		
Italian Society of Medical Oncology (AIOM) & Italian Society of Artificial Nutrition and Metabolism (SINPE)	X	X	X			X	X
Oncology/medicine							
American College of Surgeons (ACS)*	X	X	X				X
American Society of Clinical Oncology (ASCO)	X	X	X				
Association of Community Cancer Centers (ACCC)	X		X			X	X
Clinical Oncology Society of Australia (COSA)	X	X	X	X			X
European Society for Medical Oncology (ESMO)	X		X	X	X	X	
National Comprehensive Cancer Network (NCCN)	X	X	X			X	X
Gastroenterological Society of Taiwan		X	X				
United Kingdom National Multidisciplinary Guidelines	X	X	X	X	X	X	X
Exercise							
American College of Sports Medicine (ACSM)				X			
Exercise for People with Cancer Guideline Development (Cancer Care Ontario’s Program in Evidence-Based Care)				X			

^*^From Optimal Resources for Cancer Care, 2020 Standards; These standards are intended solely as qualification criteria for Commission on Cancer (CoC) accreditation. They do not constitute a standard of care and are not intended to replace the medical judgment of the physician or healthcare professional in individual circumstances

### Screening for malnutrition risk and low muscle mass

All clinical nutrition societies and several oncology societies recommend screening for malnutrition risk at diagnosis and during and after treatment (Supplementary Table 1). Several validated screening tools are available, such as the Malnutrition Screening Tool (MST), which can be administered quickly by nursing staff [33].

Screening is not mandated in most countries, nor is it standardized. Trujillo et al. reported that 53% of outpatient cancer centers in the USA screened for malnutrition risk and 65% used a validated screening tool [32]. A quality assurance performance improvement program, implemented in outpatient cancer treatment centers to assess the feasibility of standardizing malnutrition screening with a validated tool embedded in the electronic health record/electronic medical record (EHR/EMR), found that the rate of screening increased from 60% at baseline to 78% at 20 months [34].

Because muscle loss is common, the Clinical Oncology Society of Australia recommends all patients be screened for low muscle mass at diagnosis and re-screened when patients’ clinical situations change, using the SARC-F questionnaire alone or in combination with calf circumference (SARC-CalF) [35]. However, these tools have not been fully explored and validated in patients with cancer. SARC-F is a geriatric assessment related to functional outcomes and has not yet been proven to be ideal for use in patients with cancer of all ages, but may have acceptable performance among older patients with cancer [36, 37]. In the aging literature, although SARC-F performs satisfactorily for evaluating muscle function, SARC-CalF has greater screening efficacy than SARC-F for identifying low muscle function and low muscle mass in older adults [38, 39]. Importantly, adjustment factors for the confounding effects of adiposity on calf circumference have been recently published (Table 2) [40]. This study was conducted in a healthy adult population and appears to be the only feasible means to identify low calf circumference in patients with excess weight; its use in clinical practice is yet to be explored [40]. Additional data are needed to fully characterize the screening performance of SARC-CalF in oncology.

**Table.2:** BMI adjustment factors for calf circumference outside BMI range of 18.5–24.9 kg/m^2^ by ethnicity/race ( adapted from Gonzalez 2021)

	BMI category – males (cm)				BMI category – females (cm)			
	< 18.5	25–29.9	30–39.9	≥ 40	< 18.5	25–29.9	30–39.9	≥ 40
Non-Hispanic White	+ 5.0	− 3.0	− 7.0	− 12.0	+ 4.0	− 3.0	− 7.0	− 12.0
Non-Hispanic Black	+ 4.0	− 3.0	− 7.0	− 12.0	+ 4.0	− 3.0	− 7.0	− 12.0
Mexican American	+ 4.0	− 3.0	− 6.0	− 12.0	+ 4.0	− 3.0	− 6.0	− 12.0
Other	+ 3.0	− 4.0	− 7.0	− 12.0	+ 4.0	− 3.0	− 7.0	− 11.0

### Nutrition assessment to diagnose malnutrition

Nutrition assessment can be conducted using several available tools to evaluate nutrition status, such as the Subjective Global Assessment (SGA) and the Patient-Generated Subjective Global Assessment (PG-SGA) [41, 42]. Nutrition assessment is different from nutrition screening in that it determines the presence, severity, and causes of malnutrition and is used to plan nutrition intervention, while screening indicates the presence or absence of malnutrition risk. [43].

Nutrition societies recommend early nutrition assessment for all at-risk patients before anticancer treatments (including surgery) begin, with reassessments at regular intervals throughout the cancer trajectory (Supplementary Table 2). ESPEN also recommends dual X-ray absorptiometry, anthropometry, bioelectrical impedance analysis, or CT scans to assess muscle mass, and walking tests or dynamometers (i.e., hand-grip strength) for muscle function [29].

With the lack of consensus on the diagnostic criteria for malnutrition, a framework such as the one proposed by the Global Leadership Initiative on Malnutrition (GLIM) can be used to help diagnose malnutrition through (1) identifying at-risk patients using any validated screening tool and (2) conducting a nutrition assessment using validated assessment tools to diagnose and grade the severity of malnutrition. GLIM’s diagnostic criteria include three phenotypic criteria (involuntary weight loss, low BMI, and reduced muscle mass) and two etiologic criteria (reduced food intake or assimilation and inflammation or disease burden) [44].

### Interventions

#### Nutrition therapy is essential in cancer care

**Fig. 2:**
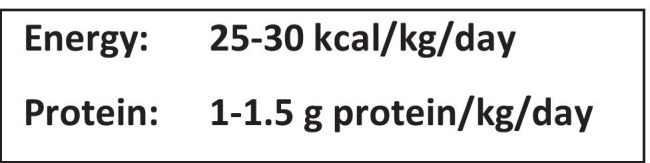
Expert energy and protein recommendations for patients with cancer

Nutrition intervention encompasses nutrition counseling and education, oral nutritional supplements (ONS), and enteral and/or parenteral nutrition support, as appropriate for each individual case. Healthcare providers should proactively identify early indicators of malnutrition risk in their patients (e.g., anorexia and reduced food intake) and intervene with additional protein and energy before cachexia develops, rather than reactively doing so when patients become severely depleted (i.e., refractory cachexia). Intervening early and throughout treatment may help to improve nutrition status and, ultimately, quality and length of life [45]. Nutrition societies recommend intervening early to support an adequate intake during and after treatment that is based on a patient’s total energy and protein needs (Supplementary Table 3 and Fig. 2), disease status, current intake, lifestyle, and food preferences. Several RCTs, systematic reviews, and meta-analyses involving patients with different tumor types and undergoing various anticancer treatments have shown that nutrition therapy improves weight status, energy and protein intakes, treatment tolerance, and survival and reduces nutrition impact symptoms, hospital readmissions, and mortality [12–15, 46]. While not all evidence supporting oncology nutrition is based on RCTs, the body of evidence is substantial and should not be dismissed. This is an important consideration given the frequency of non-evidence-based medical practice in oncology, which has been reported to occur in 33% of patients [47].

Specific nutrients may have important roles in improving nutrition status while mitigating metabolic changes and the consequential decline in muscle mass and physical function in patients with cancer. One example is eicosapentaenoic acid (EPA), a long-chain omega-3 fatty acid (ω–3) found in fish oil, with some current guidelines recommending EPA or fish oil supplementation to stabilize or improve appetite, and increase food intake, muscle mass, and body weight in patients with cachexia and/or advanced cancer who are undergoing chemotherapy [29, 48]. A systematic review and meta-analysis of 11 RCTs (*n* = 1350) demonstrated consuming high-protein ONS enriched with ω–3 fatty acids was associated with improved body weight, attenuated lean mass loss, and improved selected domains of quality of life among patients receiving chemotherapy [49].

### Exercise is important in cancer care

**Fig. 3:**
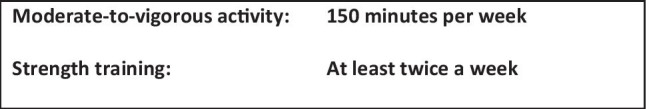
Expert recommendations on type and amount of exercise per week for patients with cancer

Aerobic and resistance exercise during anticancer treatments preserves or improves aerobic capacity, muscle mass and strength, and quality of life [50, 51]. Several societies advocate regular exercise before, during, and after treatment as a standard of care in oncology (Supplementary Table 4). Exercise is safe and well-tolerated by most patients with various cancer types [52]. However, patients who have had lung or abdominal surgeries or have ostomies, ataxia, extreme fatigue, severe malnutrition, or bone metastases need to be under the care of physical and occupational therapists, or physical medicine and rehabilitation physicians  (in conjunction with the dietitian), and should only undertake the physical activity with their guidance and supervision [52]. Exercise recommendations for most patients with cancer and healthy adults (Fig. 3) are similar, although care must be taken to support the additional energy needs of patients with malnutrition who engage in exercise [53, 54].

Prehabilitation exercise is a structured intervention implemented before surgery, which has been found to be safe, and is often delivered as a combination of moderate, continuous cardiovascular activity in combination with resistance training or high-intensity interval training, with the aim of improving functional capacity and increasing muscle mass pre-surgery or pre-treatment (e.g., within 4 to 6 weeks). Both unimodal (exercise only) and multimodal prehabilitation (exercise with nutrition) programs can facilitate recovery and reduce postoperative complications [55].

### Multidisciplinary teams provide multimodal treatment

Multidisciplinary teams are dedicated to developing and providing multimodal, patient-centered care throughout the cancer journey (Fig. 1 and Supplementary Table 5). In a pilot trial (*n* = 34) integrating a patient-centered, best-practice (i.e., evidence-based) head and neck cancer care model with an MDT approach, malnutrition screening increased from 14 to 88%, and early access to dietitians from 20 to 97%, while unplanned hospital admissions and costs were reduced, and communication and care coordination improved [16]. Of note, oncology nurse navigators, who practice mainly in the USA, can play an important role in the MDT by working closely with patients to ensure improved access to supportive care resources and optimal patient comprehension of the diagnosis and cancer treatment options.

Multimodal therapy is the combination of two or more interventions designed to improve specific outcomes (Supplementary Table 6). Despite the lack of standards to treat cachexia, evidence indicates multimodal therapy including nutrition counseling and ONS to promote protein and energy balance, EPA supplementation and non-steroidal anti-inflammatory drugs to reduce inflammation, and moderate resistance exercise to increase anabolism [56–58] will improve outcomes. Current evidence suggests that this type of multimodal intervention is feasible, safe, and associated with improvements in weight and nutrition status, physical performance, and symptom severity and may be beneficial to patients with cachexia and advanced cancers [15, 59]. The Multimodal Intervention Exercise, Nutrition and Inflammatory Medication (MENAC) is an ongoing, international RCT evaluating the effect of an early and sustained multimodal program on changes in body weight, muscle mass, and physical activity in patients newly diagnosed with lung or pancreatic cancer and starting cancer treatment (*n* = 240) [60].

Another multimodal approach implements a prehabilitation intervention that combines (1) personalized nutrition counseling and supplementation as needed, (2) individualized aerobic and weight resistance exercises, and (3) anxiety reduction and relaxation strategies to prepare patients for the anticipated detrimental effects of surgery. The majority of research has been conducted in surgery patients (94%), with several RCTs showing prehabilitation incorporating these three components facilitated earlier return to pre-surgery physical function levels and was associated with greater gains in muscle mass (compared to rehabilitation), and reduced hospital LOS and healthcare costs [61–65].

### Monitoring occurs throughout a patient’s journey

Nutrition monitoring should begin at diagnosis and continue throughout the cancer trajectory. It involves evaluating patients’ response to nutrition and exercise interventions, regularly reassessing nutrition status, and providing follow-up care to support recovery from the detrimental effects of treatment on body composition, physical function, and quality of life (Supplementary Table 7). Along with proactive nutrition and exercise interventions during cancer treatment, continuous monitoring and intervening after treatment can facilitate recovery from anticancer treatments and surgery and improve nutrition status, muscle mass, and physical function. Ensuring the continuity and monitoring of nutrition care is essential, including the transition from hospital to home [66].

### Position of the experts on nutrition care for patients with cancer

**Fig. 4:**
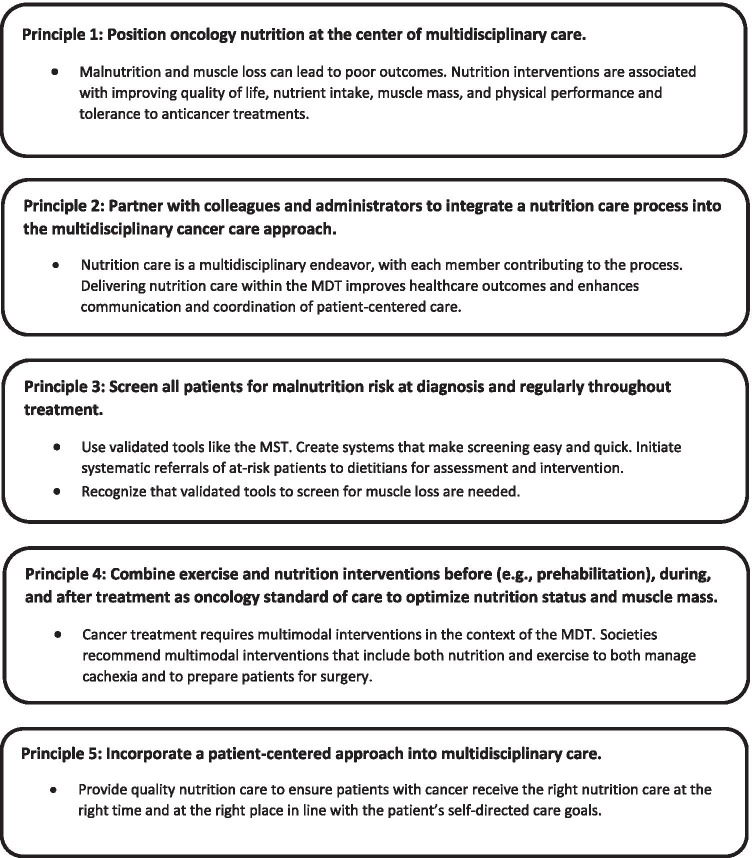
Clinical practice principles for the nutrition care of patients with cancer

Recognizing the challenges of caring for patients with cancer, and optimizing patient outcomes with nutrition and exercise interventions, this expert panel recommends combining societies’ recommendations and current evidence to implement the following principles to guide clinical practice (Fig. 4):

1. ***Position oncology nutrition at the center of multidisciplinary care***
  Ample evidence supports that adequately nourished patients benefit from anticancer treatments and supportive interventions, which lessens the challenges of medical management of this population. One path to positioning oncology nutrition as a standard of care is to ensure that MDT members are educated and trained to screen, evaluate, and monitor at-risk and malnourished patients with cancer and understand how and when to refer to a registered dietitian for more in-depth nutrition care. Most healthcare providers do not receive clinical nutrition education, and it may be low on the list of competing clinical priorities. Some societies, such as ASPEN and ESPEN, provide funding for nutrition fellowships for physicians. Perhaps extending similar educational and training opportunities to nurses would help to elevate the value and importance of oncology nutrition as standard practice within MDTs.
2. ***Partner with colleagues and administrators to integrate a nutrition care process into the multidisciplinary cancer care approach.***
  In most oncology clinics, nutrition care is usually the domain of dietitians and is managed in isolation. Delivering effective nutrition care is possible when MDT members coordinate and communicate nutrition care (Fig. 1). In addition, patients often receive conflicting information and advice about nutrition from healthcare providers; therefore, all members of the MDT need to be knowledgeable about oncology nutrition so they can provide patients with consistent and accurate nutrition guidance.
3. ***Screen all patients for malnutrition risk at diagnosis and regularly throughout treatment***
  Nursing-led screening for malnutrition at diagnosis is essential. Organizational processes to standardize screening are recommended, where positive results are documented and communicated to the MDT and patients are referred to a dietitian, as appropriate, for further assessment and follow-up.
  Although evidence indicates a high percentage of patients with newly diagnosed cancer have low muscle mass, no validated tools to screen for muscle loss currently exist, representing knowledge gaps researchers and expert societies need to address.
4. ***Combine exercise and nutrition interventions before (e.g., prehabilitation), during, and after treatment as oncology standard of care in oncology to optimize nutrition status and muscle mass***
  Cancer is a multifaceted disease requiring multimodal interventions best delivered by MDTs. Current guidelines recommend exercise combined with nutrition interventions as a standard of care. Combining these supportive interventions is associated with improving patients’ quality of life and health-related outcomes in cachexia. Perioperative multimodal interventions, such as prehabilitation, can improve physical function, increase muscle mass, and reduce complications among patients undergoing surgery.
5. ***Incorporate patient-centered approaches into multidisciplinary care***
  In patient-centered, collaborative, and coordinated care, a patient’s health needs and desired health outcomes guide decisions regarding their healthcare. The key components of patient-centered care include patient education and empowerment, patient-centered communication, coordinated and integrated care, and provision of emotional support [67].

## Conclusion/relevance

Malnutrition, muscle loss, and cachexia in cancer impact patient and healthcare outcomes. Using multimodal supportive interventions that include nutrition and exercise are associated with preventing and treating these comorbidities and improving outcomes. Delivering this level of care is achievable when societies’ recommendations become part of standard clinical practice and an MDT is involved. Early and systematic screening for malnutrition risk using validated tools and EHR/EMR technology when available is quick, feasible, and sustainable, with at-risk patients promptly referred to dietitians for assessment and intervention. The MDT approach to nutrition care in oncology is associated with improved screening, assessment, access to comprehensive care and enhances communication and collaboration within the MDT, thus improving patient care overall. Patient-centered nutrition care is at the core of MDT and is an important strategy to improve nutrition status and maintain quality of life for all patients with cancer.

## Supplementary Information

Below is the link to the electronic supplementary material.

Supplementary file1 (DOCX 26 KB)

Supplementary file2 (DOCX 27 KB)

Supplementary file3 (DOCX 34 KB)

Supplementary file4 (DOCX 25 KB)

Supplementary file5 (DOCX 24 KB)

Supplementary file6 (DOCX 25 KB)

Supplementary file7 (DOCX 24 KB)

## Data Availability

N/A.

## Code availability

N/A.
