# Supplementary material for: Examining guidelines and new evidence in oncology nutrition: a position paper on gaps and opportunities in multimodal approaches to improve patient care
Source: Support Care Cancer. 2021 Nov 23;30(4):3073–83. doi: 10.1007/s00520-021-06661-4 (PMC8857008; doi:10.1007/s00520-021-06661-4)
Supplement: Supplementary file 1 — Supplementary file1 (DOCX 26 KB) [file 520_2021_6661_MOESM1_ESM.docx]

**Table 1: Malnutrition risk screening recommendations**

| Recommendations | Society |
| --- | --- |
| Adult oncology patients should be screened using a malnutrition screening tool validated in the setting in which the tool is intended for use.  All patients with cancer should be screened on entry into oncology services; rescreening should be repeated throughout treatment to facilitate referral as needed. | Academy of Nutrition and Dietetics (AND) |
| Patients with cancer are nutritionally at risk and should undergo nutrition screening to identify those who require formal nutrition assessment with  development of a nutrition care plan. | American Society for Parenteral and Enteral Nutrition (ASPEN) |
| Screen for malnutrition, weight loss, body mass index (BMI) (as part of the geriatric assessment). | American Society of Clinical Oncology (ASCO) |
| Screen patients at risk for malnutrition. | Association of Community Cancer Centers (ACCC) (USA) |
| All people with cancer should be screened for malnutrition and sarcopenia in all health settings at diagnosis and as the clinical situation changes throughout treatment and recovery. | Clinical Oncology Society of Australia (COSA) |
| Identifying patients at risk of simple starvation or pre-cachectic patients is within the clinical expertise of oncologists. | European Society for Medical Oncology (ESMO) |
| To detect nutritional disturbances at an early stage, we recommend to regularly evaluate nutritional intake, weight change, and BMI, beginning with cancer diagnosis and repeated depending on the stability of the clinical situation.  We recommend to routinely screen all patients with advanced cancer for inadequate nutritional intake, weight loss, and low BMI, and if found at risk, to assess these patients further for both treatable nutrition impact symptoms and metabolic derangements.  For all cancer patient undergoing either curative or palliative surgery, we recommend management within an enhanced recovery after surgery (ERAS) program; within this program, every patient should be screened for malnutrition and if deemed at risk, given additional nutritional support. | European Society for Clinical Nutrition and Metabolism (ESPEN)* |
| All patients should be evaluated for nutritional risks. | National Comprehensive Cancer Network (NCCN) - USA |
| Nutritional screening should be performed using validated tools (NRS 2002 [Nutritional Risk Screening (2002)], MUST [Malnutrition Universal Screening Tool], MST [Malnutrition Screening Tool], MNA [Mini Nutritional Assessment]) upon diagnosis and systematically repeated at regular time points in patients with cancer type, stage or treatment potentially affecting nutritional status. | Italian Society of Medical Oncology (AIOM) & Italian Society of Artificial Nutrition and Metabolism (SINPE) |
| Patients with head and neck cancer should be nutritionally screened using a validated screening tool at diagnosis and then repeated at intervals through each stage of treatment. | United Kingdom National Multidisciplinary  Guidelines |
| Components of oncology nutrition services include but are not limited to: screening and nutrition assessment for risk and diagnosis of malnutrition, nutrition-related problems, and overweight and obesity. | American College of Surgeons (ACS)** |

*: These guidelines have been officially endorsed by the European Society of Surgical Oncology (ESSO), the European Association for Palliative care (EAPC) and the Chinese Society of Clinical Oncology (CSCO).

**From Optimal Resources for Cancer Care, 2020 Standards; These standards are intended solely as qualification criteria for Commission on Cancer (CoC) accreditation. They do not constitute a standard of care and are not intended to replace the medical judgment of the physician or health care professional in individual circumstances.
