# Supplementary material for: Examining guidelines and new evidence in oncology nutrition: a position paper on gaps and opportunities in multimodal approaches to improve patient care
Source: Support Care Cancer. 2021 Nov 23;30(4):3073–83. doi: 10.1007/s00520-021-06661-4 (PMC8857008; doi:10.1007/s00520-021-06661-4)
Supplement: Supplementary file 2 — Supplementary file2 (DOCX 27 KB) [file 520_2021_6661_MOESM2_ESM.docx]

**Table 2: Nutritional assessment recommendations**

| Recommendations | Society |
| --- | --- |
| Registered dietitians/nutritionists (RDNs) should use an assessment tool validated in the setting in which the tool is intended for use as part of the complete nutrition assessment (SGA [Subjective Global Assessment] and PG-SGA [Patient-Generated Subjective Global Assessment]).  Include food, beverage and nutrient intake and related history; biochemical data, medical tests, and procedures; anthropometric measurements; nutrition-focused physical findings (e.g., low muscle mass, loss of subcutaneous fat, changes in appetite, functional parameters).  In patients with lung, pancreatic and gastrointestinal cancers, and those at high risk for weight loss, RDNs should assess for nutrition impact symptoms, markers of inflammation, and other signs that may indicate (pre-)cachexia.  Use clinical judgment in interpreting nutrition assessment data to diagnose malnutrition. | Academy of Nutrition and Dietetics (AND) |
| For weight loss that is > 10% from baseline: referral to nutritionist/dietitian (geriatric oncology).  Clinicians may refer patients with advanced cancer and loss of appetite and/or body weight to a dietitian for assessment (cachexia). | American Society of Clinical Oncology (ASCO) |
| Nutrition assessment is recommended in patients who are nutritionally at risk. | American Society for Parenteral and Enteral Nutrition (ASPEN) |
| All people with cancer identified as being “at risk” of malnutrition following screening or with a cancer diagnosis or treatment plan known to lead to high risk of malnutrition should have comprehensive nutrition assessment using a tool validated in the oncology population.  All people with cancer identified as being “at risk” of sarcopenia following appropriate screening should have a comprehensive evaluation of muscle status using a combination of assessments for muscle mass, muscle strength and function. | Clinical Oncology Society of Australia (COSA) |
| In patients with abnormal screening, we recommend objective and quantitative assessment of nutritional intake, nutrition impact symptoms, muscle mass, physical performance and the degree of systemic inflammation. | European Society for Clinical Nutrition and Metabolism (ESPEN)* |
| Assess nutritional status before and after major surgery. | ESPEN (Surgery) |
| If screening identifies a patient at risk, a formal and extensive nutritional assessment should be performed by an appropriately trained professional. | ESPEN (Perioperative nutrition) |
| Nutritional assessment at the diagnosis, prior to definitive therapy and a change of treatment strategy are suggested.  Time interval for nutrition assessment can range from two weeks during treatment to one month when stability is achieved after completion of treatment. | Gastroenterological Society of Taiwan |
| Patients at nutritional risk should be promptly referred for comprehensive  nutritional assessment and support to clinical nutrition services or medical  personnel with documented skills in clinical nutrition, specifically for cancer  patients. | Italian Society of Medical Oncology (AIOM) & Italian Society of Artificial Nutrition and Metabolism (SINPE) |
| Nutritional status of patients should be evaluated pre- and post-treatment using objective and subjective assessment tools. | National Comprehensive Cancer Network (NCCN) - USA |
| Perform nutritional assessment frequently, including pre-treatment.  Use validated tools (e.g., PG-SGA or SGA) to assess nutritional status.  Offer pre-treatment assessment prior to any treatment as intervention aims to improve, maintain or reduce decline in nutritional status of head and neck cancer patients who have malnutrition or are at risk of malnutrition.  Patients identified as well-nourished at baseline but whose treatment may impact on their future nutritional status should receive dietetic assessment and intervention at any stage of the pathway.  Quality of life parameters including nutritional and swallowing, should be measured at diagnosis and at regular intervals post-treatment. | United Kingdom National Multidisciplinary  Guidelines |
| Components of oncology nutrition services include but are not limited to: screening and nutrition assessment for risk and diagnosis of malnutrition, nutrition-related problems, and overweight and obesity. | American College of Surgeons (ACS)** |

*: These guidelines have been officially endorsed by the European Society of Surgical Oncology (ESSO), the European Association for Palliative care (EAPC) and the Chinese Society of Clinical Oncology (CSCO).

**From Optimal Resources for Cancer Care, 2020 Standards; These standards are intended solely as qualification criteria for Commission on Cancer (CoC) accreditation. They do not constitute a standard of care and are not intended to replace the medical judgment of the physician or health care professional in individual circumstances.
