# Supplementary material for: Examining guidelines and new evidence in oncology nutrition: a position paper on gaps and opportunities in multimodal approaches to improve patient care
Source: Support Care Cancer. 2021 Nov 23;30(4):3073–83. doi: 10.1007/s00520-021-06661-4 (PMC8857008; doi:10.1007/s00520-021-06661-4)
Supplement: Supplementary file 3 — Supplementary file3 (DOCX 34 KB) [file 520_2021_6661_MOESM3_ESM.docx]

**Table 3: Nutritional intervention recommendations**

| Recommendations | Society |
| --- | --- |
| Assesses patient's and/or family's ability to understand and comply with nutritional education and instructions and modifies interventions appropriately  Provides anticipatory guidance, identifying common nutritional problems the patient may encounter during the course of treatment  Addresses side-effect management in the context of evidence-based nutrition care, and services across the care continuum (prevention, treatment, survivorship, palliative care, and hospice) | Association of Community Cancer Centers (ACCC) (USA) |
| If an adult oncology patient is undergoing chemotherapy or radiation therapy, RDNs should provide medical nutrition therapy (MNT).  If suboptimal symptom control or inadequate dietary is an issue and patient is still experiencing loss of weight and lean mass, RDN may consider eicosapentaenoic (EPA)-enriched oral nutritional supplements (ONS).  If an adult oncology patient has neutropenia, RDNs should provide dietary counseling on safe food handling and foods that may pose infectious risks during the period of neutropenia.  Patients with pre-cachexia or cancer cachexia should receive prompt and aggressive intervention to address nutrition impact symptoms and preserve or prevent loss of lean mass and weight. | Academy of Nutrition and Dietetics (AND) - EAL |
| Assess need for meal preparation and institute support interventions if necessary (e.g., caregiver and Meals-on-Wheels).  Clinicians may refer patients with advance cancer and loss of appetite and/or body weight to Registered Dietitian (RD) for counseling, with goals of providing patients/caregivers with practice and safe advice for feeding.  Education on: high protein, high-calorie, nutrient-dense foods  Enteral nutrition (EN) should not be routinely offered to manage cancer cachexia in patients with advanced cancer.  Discontinuing previously initiated EN/parenteral nutrition (PN) is appropriate near end of life  PN should not be routinely offered to manage cancer cachexia in patients with advanced cancer.  Short-term trial of PN may be offered to a select group of patients, e.g., those who have reversible bowel obstruction, short bowel syndrome (SBS), or other issues contributing to malabsorption, but otherwise are reasonably fit. | American Society of Clinical Oncology (ASCO) |
| Patients should receive dietary counseling regarding foods which may pose infectious risks and safe food handling during the period of neutropenia.  Perioperative nutrition support therapy (NST) may be beneficial in moderately/severely malnourished pts if administered 7-14 preoperatively; potential benefits of NST must be weighed against potential risks of NST and of delaying the operation.  NST is appropriate for patients receiving active anticancer treatment /undergoing hematopoietic cell transplantation who are malnourished and who are anticipated to be unable to ingest and/or absorb adequate nutrients for a prolonged period of time.  n-3 fatty acid supplementation may help stabilize weight in pts on oral diets experiencing progressive, unintentional weight loss.  Immunonutrition EN may be beneficial in patients undergoing major cancer operations. | American Society for Parenteral and Enteral Nutrition (ASPEN) |
| All people with cancer-related malnutrition and sarcopenia should have access to the core components of treatment including MNT. | Clinical Oncology Society of Australia (COSA) |
| Patients should have optimal nutritional care irrespective of tumor type and throughout the course of their disease. Initial aim should be to work with the patient to provide macro- and micronutrients sufficient to prevent further involuntary weight loss.  Efforts should be made to provide adequate nutrition (along with exercise) to preserve muscle mass and reduce systemic inflammation intervene early and throughout treatment.  Nutrition support and counseling as part of a patient-centered package of supportive measures. | European Society for Medical Oncology (ESMO) |
| We recommend that the total energy expenditure (TEE) of cancer patients, if not measured individually, be assumed to be similar to healthy subjects and generally ranging between 25 and 30 kcal/kg/day.  We recommend that protein intake should be above 1 g/kg/day and, if possible up to 1.5 g/kg/day.  We recommend that vitamins and minerals be supplied in amounts approximately equal to the recommended daily allowance and discourage the use of high-dose micronutrients in the absence of specific deficiencies.  In weight-losing cancer patients with insulin resistance, we recommend to increase the ratio of energy from fat to energy from carbohydrates. This is intended to increase the energy density of the diet and to reduce the glycemic load.  We recommend nutritional intervention to increase oral intake in cancer patients who are able to eat but are malnourished or at risk of malnutrition. This includes dietary advice, the treatment of symptoms and derangements impairing food intake (nutrition impact symptoms) and offering ONS.  We recommend not to use dietary provisions that restrict energy intake in patients with or at risk of malnutrition.  If a decision has been made to feed a patient, we recommend EN if oral nutrition remains inadequate despite nutritional interventions (counseling, ONS), and PN if EN is not sufficient or feasible.  If oral food intake has been decreased severely for a prolonged period, we recommend to increase (oral, enteral or parenteral) nutrition only slowly over several days and to take additional precautions to prevent a refeeding syndrome.  In patients with chronic insufficient dietary intake and/or uncontrollable malabsorption, we recommend home EN or PN in suitable patients.  For all cancer patients undergoing either curative or palliative surgery, we recommend management within an enhanced recovery after surgery (ERAS) program; within this program, every patient should be screened for malnutrition and if deemed at risk, given additional nutritional support.  In surgical cancer patients at risk of malnutrition or who are already malnourished, we recommend appropriate nutritional support both during hospital care and following discharge from the hospital.  In upper gastrointestinal (GI) cancer patients undergoing surgical resection in the context of traditional perioperative care, we recommend oral/enteral immunonutrition (arginine, n-3 fatty acids, nucleotides).  We recommend that during radiotherapy – with special attention to radiotherapy of the head and neck, thorax and GI tract – an adequate nutritional intake should be ensured primarily by individualized nutritional counseling and/or with use of ONS, in order to avoid nutritional deterioration, maintain intake and avoid radiotherapy interruptions.  We recommend to screen for and manage dysphagia and to encourage and educate patients on how to maintain their swallowing function during EN.  We recommend enteral feeding using nasogastric or percutaneous tubes (e.g. percutaneous endoscopic gastrostomies [PEG]) in radiation-induced severe mucositis or obstructive tumors of the head-neck or thorax.  We do not recommend PN as a general treatment in radiotherapy but only if adequate oral/EN is not possible, e.g. in severe radiation enteritis or severe malabsorption.  There are insufficient consistent clinical data to recommend glutamine to prevent radiation-induced enteritis/diarrhea, stomatitis, esophagitis, or skin toxicity.  There are insufficient consistent clinical data to recommend probiotics to reduce radiation-induced diarrhea.  In a patient undergoing curative anticancer drug treatment, if oral food intake is inadequate despite counseling and ONS, we recommend supplemental EN or, if this is not sufficient or possible, PN.  During intensive chemotherapy and after stem cell transplantation we recommend maintaining physical activity and to ensure an adequate nutritional intake. This may require EN and/or PN.  If oral nutrition is inadequate we suggest preferring EN to PN, unless there is severe mucositis, intractable vomiting, ileus, severe malabsorption, protracted diarrhea or symptomatic GI graft versus host disease.  There are insufficient consistent clinical data to recommend glutamine to improve clinical outcome in patients undergoing high-dose chemotherapy and hematopoietic stem cell transplantation (HSCT).  In cancer survivors, we recommend maintaining a healthy weight (BMI 18.5-25 kg/m^2^) and to maintain a healthy lifestyle, which includes being physically active and a diet based on vegetables, fruits, and whole grains and low in saturated fat, red meat, and alcohol. | European Society for Clinical Nutrition and Metabolism (ESPEN) |
| Perioperative nutritional therapy is indicated in patients with malnutrition and those at nutritional risk. Perioperative nutritional therapy should also be initiated, if it is anticipated that the patient will be unable to eat for more than five days perioperatively. It is also indicated in patients expected to have low oral intake and who cannot maintain above 50% of recommended intake for more than seven days. In these situations, it is recommended to initiate nutritional therapy (preferably by the enteral route ONS-TF) without delay.  If the energy and nutrient requirements cannot be met by oral and enteral intake alone (< 50% of caloric requirement) for more than seven days, a combination of EN and PN is recommended. PN shall be administered as soon as possible if nutrition therapy is indicated and there is a contraindication for EN, such as in intestinal obstruction. | ESPEN (Surgery) |
| Patients identified as high risk of malnutrition should be evaluated for preoperative EN for at least 7-10 days.  EN is preferable to PN.  EN immunonutrition may preserve lean mass and attenuate stress response post-esophagostomy vs. standard EN.  Immunomodulating EN may achieve better nutritional status or maintain immune function during concurrent chemotherapy.  Consider PN only when other routes of administration are impractical or not feasible. | Gastroenterological Society of Taiwan (Esophageal Cancer) |
| Nutritional support should be actively managed and targeted for each patient according to nutritional conditions, clinical status, planned treatment and expected outcome. It should comprise nutritional counseling with the possible use of ONS and/or artificial nutrition (EN and/or PN) according to spontaneous food intake, tolerance and effectiveness.  Nutritional support and dietary modifications should aim to assist the maintenance or recovery of nutritional status by increasing or preserving protein and calorie intake. “Alternative hypocaloric anticancer diets” (e.g. macrobiotic or vegan diets) are not recommended.  Nutritional support may be integrated into palliative care programs, according to individual-based evaluations, quality of life implications, life expectancy and patients’ awareness.  Home artificial nutrition should be prescribed and regularly monitored  using defined protocols shared between oncologists and clinical nutrition  specialists. | Italian Society of Medical Oncology (AIOM) & Italian Society of Artificial Nutrition and Metabolism (SINPE) |
| All patients should receive dietary counseling with initiation of anticancer treatment.  EN tube feeding via PEG or nasogastric (NG) tube is to be considered for patients with severe weight loss (5% weight loss over past one month; 10% weight loss over six months); insufficient nutrient intake or difficulty swallowing. | National Comprehensive Cancer Network (NCCN) - USA |
| Patients at high risk should be referred to dietitian for early intervention.  Initiate nutrition intervention early when deficits are detected.  Weekly dietetic intervention is offered for all patients undergoing radiotherapy treatment to prevent weight loss, increase intake and reduce treatments interruptions.  Start nutritional therapy if undernutrition already exists, or if it is anticipated that the patient will be unable to eat for more than seven days.  Consider gastrostomy insertion if long-term tube feeding is necessary (greater than four weeks).  Offer prophylactic tube feeding as part of locally agreed guidelines, where oral nutrition is inadequate.  Offer dietary counseling and/or ONS for up to three mos post-treatment.  Aim for energy intake > 30 kcal/kg/d post-operatively.  Aim for at least 30 kcal/kg/d and 1.2 g protein/kg/d during radiation therapy or chemotherapy.  Commence nutritional intervention with fat-free or medium chain triglyceride nutritional supplements either orally or via a feeding tube.  Start EN if malnutrition exists, or if patient is unable to eat for > seven days or if oral intake is inadequate (< 60% of estimated energy expenditure) for > 10 days; use standard polymeric feed.  Consider PN in severe cases of chyle leak when drainage volume is consistently high.  Integrate measures to modulate cancer cachexia changes into the nutritional management.  Patients who have completed their rehabilitation and are disease free should be offered healthy eating advice as part of a health and wellbeing. | United Kingdom National Multidisciplinary  Guidelines (Head & Neck) |
| Components of oncology nutrition services include, but are not limited to: MNT, nutrition counseling, nutrition education, and management of enteral and parenteral nutrition | American College of Surgeons (ACS)* |

*From Optimal Resources for Cancer Care, 2020 Standards; These standards are intended solely as qualification criteria for Commission on Cancer (CoC) accreditation. They do not constitute a standard of care and are not intended to replace the medical judgment of the physician or health care professional in individual circumstances.
