# Supplementary material for: Examining guidelines and new evidence in oncology nutrition: a position paper on gaps and opportunities in multimodal approaches to improve patient care
Source: Support Care Cancer. 2021 Nov 23;30(4):3073–83. doi: 10.1007/s00520-021-06661-4 (PMC8857008; doi:10.1007/s00520-021-06661-4)
Supplement: Supplementary file 4 — Supplementary file4 (DOCX 25 KB) [file 520_2021_6661_MOESM4_ESM.docx]

**Table 4: Exercise recommendations**

| Recommendations | Society |
| --- | --- |
| Standard exercise testing methods are generally appropriate for patients with cancer who do not require pre-exercise medical evaluation or who have been medically cleared for exercise.  Cancer survivors should receive a comprehensive assessment of all components of health-related physical fitness (i.e., cardiorespiratory fitness, muscle strength and endurance, body composition, and flexibility), with some specific cancer-specific considerations.  No assessments are required to start low-intensity aerobic training (e.g., walking or cycling), resistance training with gradual progression, or a flexibility program in most survivors.  150 minutes/week of moderate-vigorous exercise and resistance training two days/week | American College of Sports Medicine (ACSM) |
| Exercise to be embedded as part of standard practice, viewed as adjunct treatment to help counteract adverse effects of cancer and cancer treatment.  Refer pts to accredited exercise physiologist/physical therapist with experience in cancer care.  All people with cancer-related malnutrition and sarcopenia should have access to the core components of treatment including targeted exercise prescription and physical activity advice, and physical and psychological symptom management.  150 minutes/week of moderate-vigorous exercise and resistance exercise two to three days/week. | Clinical Oncology Society of Australia (COSA) |
| Efforts should be made to encourage physical exercise (within the capacities of the patient) as a means of preserving and restoring muscle mass, and to reduce inflammation (while means of doing so are not yet clear; optimal nutritional care throughout the course of [the] disease. | European Society for Medical Oncology (ESMO) |
| Clinicians should advise pts to engage in exercise consistent with the American College of Sports Medicine and the Canadian Society for Exercise Physiology:   - 150 min of moderate-intensity aerobic exercise spread over three to five days/week - Resistance training at least two days/week   Pre-exercise assessment to evaluate for effects of disease, treatment, comorbidities before starting exercise intervention  Exercise in group or supervised setting | Exercise for People with Cancer Guideline Development (Cancer Care Ontario’s Program in Evidence-Based Care) |
| We recommend maintenance or an increased level of physical activity in cancer patients to support muscle mass, physical function, and metabolic pattern.  We suggest individualized resistance exercise in addition to aerobic exercise to maintain muscle strength and muscle mass.  During intensive chemotherapy and after stem cell transplantation we recommend maintaining physical activity and to ensure an adequate nutritional intake. This may require EN and/or PN.  We recommend that cancer survivors engage in regular physical activity. | European Society for Clinical Nutrition and Metabolism (ESPEN) |
| Prehabilitation (including nutrition, exercise, and rest) is recommended to improve long-term cancer outcomes. | ESPEN (Perioperative Nutrition) |
