# Supplementary material for: Examining guidelines and new evidence in oncology nutrition: a position paper on gaps and opportunities in multimodal approaches to improve patient care
Source: Support Care Cancer. 2021 Nov 23;30(4):3073–83. doi: 10.1007/s00520-021-06661-4 (PMC8857008; doi:10.1007/s00520-021-06661-4)
Supplement: Supplementary file 5 — Supplementary file5 (DOCX 24 KB) [file 520_2021_6661_MOESM5_ESM.docx]

**Table 5: Multidisciplinary care recommendations**

| Recommendations | Society |
| --- | --- |
| Registered Dietitian Nutritionist (RDN) works with patients, families and/or caregivers, physicians and other member of the multidisciplinary team (MDT) to help maintain optimal nutritional status throughout the continuum of care (prevention, treatment, survivorship, palliative care, and hospice). | Association of Community Cancer Centers (ACCC) (USA) |
| RDNs should collaborate with other health care professionals, administrators, and public policy decision makers to ensure that the evaluation of nutritional status is a key component of the adult oncology patient care process. | Academy of Nutrition and Dietetics (AND) - EAL |
| Use defined protocols shared between oncologists and clinical nutrition specialists to monitor home artificial nutrition. | Italian Society of Medical Oncology (AIOM) & Italian Society of Artificial Nutrition and Metabolism (SINPE) |
| Treatment for cancer-related malnutrition and sarcopenia should be individualized, in collaboration with the MDT and tailored to meet needs at each stage of cancer treatment.  All members of MDT promote physical activity; recommend that people with cancer adhere to exercise guidelines. | Clinical Oncology Society of Australia (COSA) |
| Multidisciplinary expertise of the Registered Dietitian (RD) and speech-language-swallowing therapist should be utilized throughout the continuum of care. | National Comprehensive Cancer Network (NCCN) - USA |
| Specialist dietitian should be part of the MDT for treating head and neck cancer patients, as frequent dietetic contact has been shown to enhance outcomes. | United Kingdom National Multidisciplinary  Guidelines (Head & Neck) |
| The cancer program holds multidisciplinary cancer case conference(s) to evaluate patient management. Multidisciplinary physician attendance plus additional physician or non-physician specialists including genetic professionals, clinical research professionals, palliative care providers, psychosocial providers, rehabilitation providers, and supportive services. | American College of Surgeons (ACS)* |

*From Optimal Resources for Cancer Care, 2020 Standards; These standards are intended solely as qualification criteria for Commission on Cancer (CoC) accreditation. They do not constitute a standard of care and are not intended to replace the medical judgment of the physician or health care professional in individual circumstances.
