# Supplementary material for: Examining guidelines and new evidence in oncology nutrition: a position paper on gaps and opportunities in multimodal approaches to improve patient care
Source: Support Care Cancer. 2021 Nov 23;30(4):3073–83. doi: 10.1007/s00520-021-06661-4 (PMC8857008; doi:10.1007/s00520-021-06661-4)
Supplement: Supplementary file 6 — Supplementary file6 (DOCX 25 KB) [file 520_2021_6661_MOESM6_ESM.docx]

**Table 6: Multimodal intervention recommendations**

| Recommendations | Society |
| --- | --- |
| Registered Dietitian Nutritionists (RDNs) should be members of interdisciplinary teams providing multimodal therapy to adult oncology patients undergoing chemotherapy or radiation therapy. | Academy of Nutrition and Dietetics (AND) |
| Multimodal supportive can be used to mitigate metabolic alterations (cachexia): oral nutritional supplements (ONS), non-steroidal anti-inflammatory drugs (NSAIDs), n3-fatty acids, and exercise. | European Society for Medical Oncology (ESMO) |
| During anticancer drug treatment, we recommend to ensure an adequate nutritional intake and to maintain physical activity.  For a patient undergoing repeated surgery as part of a multimodal oncological pathway, we recommend the management of each surgical episode within an ERAS program.  For all cancer patients undergoing either curative or palliative surgery, we  recommend management within an enhanced recovery after surgery (ERAS) program; within this program, every patient should be screened for malnutrition and if deemed at risk, given additional nutritional support.  We suggest considering corticosteroids to increase the appetite of anorectic cancer  patients with advanced disease for a restricted period (1-3 weeks) but to be aware of side effects (e.g. muscle wasting, insulin resistance, infections).  We suggest considering progestins to increase the appetite of anorectic cancer  patients with advanced disease but to be aware of potentially serious side effects (e.g. thromboembolism).  In patients with advanced cancer undergoing chemotherapy and at risk of weight  loss or malnourished, we suggest using supplementation with long-chain N-3 fatty acids or fish oil to stabilize or improve appetite, food intake, lean body mass, and body weight.  In patients complaining about early satiety, after diagnosing and treating constipation, we suggest to consider prokinetic agents, but to be aware of potential  adverse effects of metoclopramide on the central nervous system and domperidone on cardiac rhythm.  There are insufficient consistent clinical data to recommend the supplementation  with branched-chain or other amino acids or metabolites to improve fat-free mass.  There are insufficient consistent clinical data to recommend non-steroidal anti-  inflammatory drugs to improve body weight in weight-losing cancer patients.  There are insufficient consistent clinical data to recommend cannabinoids to improve taste disorders or anorexia in cancer patients.  There are insufficient consistent clinical data to recommend currently approved  androgenic steroids to increase muscle mass. | European Society for Clinical Nutrition and Metabolism (ESPEN) |
| Prehabilitation (including nutrition, exercise, and rest) is recommended to improve long-term cancer outcomes. | ESPEN (Perioperative Nutrition) |
| Integrate measures to modulate cancer cachexia changes into the nutritional management. Management approach should be multifactorial and  includes assessment and ongoing monitoring with intensive nutritional support, anti-inflammatory treatment, symptom control as well as oncological treatment options to reduce the catabolic effect of the cancer. | United Kingdom National Multidisciplinary  Guidelines |
