# Supplementary material for: Examining guidelines and new evidence in oncology nutrition: a position paper on gaps and opportunities in multimodal approaches to improve patient care
Source: Support Care Cancer. 2021 Nov 23;30(4):3073–83. doi: 10.1007/s00520-021-06661-4 (PMC8857008; doi:10.1007/s00520-021-06661-4)
Supplement: Supplementary file 7 — Supplementary file7 (DOCX 24 KB) [file 520_2021_6661_MOESM7_ESM.docx]

**Table 7: Monitoring recommendations**

| Recommendations | Society |
| --- | --- |
| To check progress, a Registered Dietitian Nutritionist (RDN) should monitor and evaluate the following components of adult oncology patients at each visit and compare with desired individual outcomes. This may include but is not limited to anthropometric measurements; food- and nutrition-related history; biochemical data, medical tests, and procedures; nutrition focused physical findings; client history; patient/family/client medical/health history; social history; and psychological/socioeconomic issues. | Academy of Nutrition and Dietetics (AND) - EAL |
| Registered Dietitian (RD) monitors the patient’s progress and provide follow-up nutrition care and evaluates nutrition outcome indicators. | Association of Community Cancer Centers (ACCC) (USA) |
| Optimizing supportive care during successive treatments (perhaps over many years) has the potential to ameliorate the adverse effects of therapy on body composition, physical function and quality of life. | European Society for Medical Oncology (ESMO) |
| Regular nutritional monitoring using defined protocols | Italian Society of Medical Oncology (AIOM) & Italian Society of Artificial Nutrition and Metabolism (SINPE) |
| Close monitoring of nutritional status is recommended in patients with significant weight loss or difficulty swallowing in head and neck cancers  Regular follow-up with the RD should continue at least until the patient has achieved a nutritionally stable baseline following treatment. For some patients with chronic nutrition challenges, this follow-up should be ongoing.  for patients who did not warrant prophylactic PEG or NG tube placement pre-treatment, caloric intake, treatment related side effects, and tube placement if two or more criteria apply: inadequate food intake, weight loss > 5% in 1 month; severe mucositis; > 60 years  Swallowing function status should be monitored for the lifetime of the patient | National Comprehensive Cancer Network (NCCN) - USA |
| Monitor weight post-operatively as energy requirements may be elevated  Monitor weight and intake regularly to determine if energy requirements are being met during radiation and chemotherapies.  Monitor nutritional parameters throughout patient’s cancer journey.  For patients with cancer cachexia, provide ongoing monitoring. | United Kingdom National Multidisciplinary  Guidelines |
